# Phytochemical, Antioxidant, Antimicrobial and Safety Profile of *Glycyrrhiza glabra* L. Extract Obtained from Romania

**DOI:** 10.3390/plants13233265

**Published:** 2024-11-21

**Authors:** Iulia Semenescu, Stefana Avram, Diana Similie, Daliana Minda, Zorita Diaconeasa, Delia Muntean, Antonina Evelina Lazar, Daniela Gurgus, Corina Danciu

**Affiliations:** 1 Department of Pharmacognosy, “Victor Babes” University of Medicine and Pharmacy, 2nd Eftimie Murgu Square, 300041 Timisoara, Romania; iulia.semenescu@umft.ro (I.S.); diana.similie@umft.ro (D.S.); daliana.minda@umft.ro (D.M.); corina.danciu@umft.ro (C.D.); 2 Research and Processing Center for Medicinal and Aromatic Plants, “Victor Babes” University of Medicine and Pharmacy, 2nd Eftimie Murgu Square, 300041 Timisoara, Romania; 3 Department of Food Science and Technology, Faculty of Food Science and Technology, University of Agricultural Science and Veterinary Medicine, Calea Manastur, 3-5, 400372 Cluj-Napoca, Romania; zorita.sconta@usamvcluj.ro; 4 Department of Microbiology, Faculty of Medicine, “Victor Babes” University of Medicine and Pharmacy Timisoara, 2nd Eftimie Murgu Square, 300041 Timisoara, Romania; muntean.delia@umft.ro; 5 Multidisciplinary Research Center on Antimicrobial Resistance, “Victor Babes” University of Medicine and Pharmacy, 2nd Eftimie Murgu Square, 300041 Timisoara, Romania; 6 National Institute of Research and Development for Electrochemistry and Condensed Matter, 144 Dr. A. P. Podeanu, 300569 Timisoara, Romania; antonina_pop@yahoo.com; 7 Department of Balneology, Medical Recovery and Rheumatology, Family Discipline, Center for Preventive Medicine, “Victor Babes” University of Medicine and Pharmacy, 2nd Eftimie Murgu Square, 300041 Timisoara, Romania; gurgus.daniela@umft.ro

**Keywords:** licorice root extract, methanol, glycyrrhizin, phenolic compounds, antioxidant, metals, antimicrobial, HET-CAM assay

## Abstract

*Glycyrrhiza glabra* L., also known as licorice, belongs to the *Fabaceae* family and is one of the most commercially valuable plants worldwide, being used in the pharmaceutical, cosmetic, and food industries, both for its therapeutic benefits as well as for the sweetening properties of the extract. This study evaluates the phytochemical composition, the biological activities, and the safety profile of a methanolic extract of licorice root (LRE) obtained from Romania. Ten phytocompounds were quantified by the HPLC-DAD-ESI+, the most abundant being the triterpene glycyrrhizin (13.927 mg/g dry extract.), followed by these flavonoids: liquiritin, liquiritigenin-apiosyl-glucoside, and apigenin-rutinoside liquiritigenin. The total phenolic content of the LRE was found to be 169.83 mg gallic acid/g dry extract. (GAE/g d.e.), and the extract showed a maximum of 79.29% antioxidant activity in the 2,2-diphenyl-1-picrylhydrazyl (DPPH) assay. Good antimicrobial activity of the LRE was observed for Gram-negative bacteria, especially for *S. pneumoniae* and *S. pyogenes*. The mineral content of the LRE was indicative of the lack of toxicity; heavy metals such as lead, cadmium, arsenic, nickel, and cobalt were below the detection limit. The safety profile of the licorice extract was assessed using the in vivo hen egg test-chorioallantoic membrane (HET-CAM protocol), indicating no irritability, good tolerability, and biocompatibility. The phytochemical and biological characterization of the Romanian licorice root extract reveals a good source of glycyrrhizin and polyphenols with antioxidant and antimicrobial potential, along with a safety profile that may be useful for future therapeutic applications.

## 1. Introduction

Herbs have been used to provide health and medical solutions for more than 3000 years. However, throughout the last two centuries, the use of herbal remedies has expanded exponentially [1]. The global market for herbal medicines has reported an increase of around 8% in the past years and is expecting a 15% compound annual growth rate from 2023 to 2033 [2,3]. The WHO estimates that close to 80% of the world population is currently using plant-based therapies to treat primary health issues [4].

Licorice (*G. glabra* L.) is a perennial shrub belonging to the *Fabaceae* (*Leguminosae*) family. It was first described and listed by Pedanius Dioscorides in the first century AD (ca. 40–90), placing it among the 650 medicinal plants listed in *De Materia Medica* [5]. Licorice is grown commercially in Italy, Spain, Greece, France, Iran, Iraq, Turkey, Turkmenistan, Uzbekistan, Syria, Afghanistan, Azerbaijan, India, China, the United States, and England [6]. It is one of the most commercially valuable plants globally, being used in the pharmaceutical, cosmetic, and food industries, both for its therapeutic benefits as well as for the sweetening properties of the extract [6].

The rhizomes and roots of *G. glabra* L. have been used for centuries, in different types of extracts, for their anti-ulcerative, antimicrobial, expectorant, analgesic, and anti-inflammatory activities [7]. In traditional Chinese medicine, licorice received the title of “king of medicine materials”, being used for the treatment of fatigue, cough with sputum, acute pain, digestive problems, liver disorder, arthritis, carbuncles, or palpitations [8,9]. More recent pharmacological studies confirmed the many traditional uses of licorice and added the following new properties: free radical scavenging [10,11], antibacterial [11,12], antidiabetic [13], antitussive and expectorant [14,15], anti-inflammatory, antiviral, immunomodulator [14,15], skin protector [6], and anti-carcinogenic [10,16].

The *G. glabra* L. complex composition has been extensively investigated, especially in recent years. Zhang and Ye [17] found more than 400 compounds within the *Glycyrrhiza* species, among which are flavonoids, phenols, triterpenes, anthraquinones, saponins, and essential oils. The saponins are the major constituents of licorice. They are mostly present in the underground parts of the plant, with glycyrrhizin (glycyrrhizic acid) being the most significant representative and is responsible for the sweet taste [14]. Glycyrrhizin represents approximately 10% of the dry root weight (depending on the pedoclimatic conditions and type of extract) [15]. From a therapeutical point of view, glycyrrhizin is the main triterpenoid saponin, showing significant anti-inflammatory, antioxidant, immunoregulatory, and antiviral effects [18]. The metabolic processes that take place throughout the plant hydrolyze glycyrrhizin into two pentacyclic triterpenic stereoisomers: 18α- and 18β-glycyrrhetinic acids [19]. Because of their chemical structure, these derivatives present great potential for biological activity [19]. Other saponins identified in the licorice root were glabasaponins A–G [20], 30-hidroxyglycyrrhizin [21], urlasaponins, and 22b-acetoxyl-glycyrrhizin [22]. Many of the other biologically active compounds identified are present throughout the entire plant (including the rhizomes) and account (in total) for 40–50% of the total dry weight [14].

The production of ROS (reactive oxygen species) is a normal part of the body’s metabolism [23]. They are involved in many different activities, such as mediating the immune response or preserving cellular homeostasis [24]. However, the overproduction of ROS results in other unfavorable processes that ultimately lead to various diseases [23,24]. Antioxidants have the ability to protect the human body from these processes. Out of all the compounds that can be found in plants, flavonoids are known to exhibit the most antioxidant properties [24].

Licorice root has been intensively investigated for its significant flavonoid content. The flavonoids identified belong to different classes, including flavanones, flavones, flavanonols, chalcones, isoflavans, isoflavenes, isoflavones, and isoflavanones. Among them, the glycosides of liquiritigenin are the major constituents, presenting good antioxidant, antimicrobial, and anti-inflammatory effects [18]. The significant antioxidant properties are also provided by the isoflavones glabridin and hispaglabridins A and B [14].

Besides its known antioxidant activity, licorice is also reported to have important antibacterial activity [25,26]. There are several studies that show the ability of different licorice extracts to inhibit the growth of both Gram-negative and Gram-positive bacteria, such as *E. coli*, *S. aureus*, and *B. subtilis* [25,26,27,28].

Furthermore, it has been shown that the rhizomes of certain species (Glycyrrhiza species being part of them) can store great amounts of the chemicals present in the soil [29]. Most of these plants are able to attenuate the harmful effect that some of these minerals may present, only if they are present in “regular” amounts [30]. On the other hand, some minerals are a normal part of the physiology of plants; trace elements are beneficial to humans, playing both preventive and curative roles. However, the accumulation of heavy metals in plants, for any reason, may pose a health risk for the human body, as the concentration range between deficiency and toxicity levels is narrow [31].

In 1989, the WHO started regulating the maximum amount of toxic metals present in finished medicinal products. They recommend that all medicinal plants that will be used as raw materials are to be checked for the presence of heavy metals [32]. Because of these regulations, studies related to the phytochemical composition of plant extracts started using different approaches and techniques to properly identify potential heavy metals. This was conducted to assess the purity, safety, and efficacy of the extracts [31]. Additionally, a plant extract intended for further use in bio-medical applications also needs a safety assessment performed in vivo. For this purpose, the chorioallantoic membrane (CAM) assay is considered an in vivo experimental alternative to animal models, with advantages such as time efficiency, accessibility, and lower costs. More specifically, to estimate the biocompatibility and the irritation potential of natural products, the hen egg test chorioallantoic membrane assay (HET-CAM) can be employed, offering characteristics regarding the effect of plant extracts upon the vascular functionality of epithelial tissues [33].

This study was designed as a comprehensive evaluation of the methanolic extract of *G. glabra* L. root cultivated in Romania. There are limited data available describing the chemical composition and biological activities of licorice root obtained from Romania. Consequently, our focus was to assess the total phenolic content (TPC) and the LC-MS phytochemical profile of the obtained licorice root extract. A screening of the inorganic elements, including heavy metals, was also achieved. Subsequently, to provide a biological characterization of the licorice root extract, the antioxidant and the antibacterial activity were evaluated in vitro, followed by the irritative potential investigation of the LR extract using the in vivo HET-CAM assay.

## 2. Results

### 2.1. Phytochemical Profile of the G. glabra Extract

A total of nine phenolic compounds (six flavones, three flavanones—Table 1) and one triterpenoid saponin were identified in the methanolic extract of *G. glabra*. Glycyrrhizin, a triterpenic compound considered to be the main component in the *G. glabra* roots, had a concentration of 13.927 mg/g. Among the total amount of identified phenolic compounds (14.208 mg/g), the highest concentrations were represented by liquiritin (5.037 mg/g), followed by liquiritigenin-apiosyl-glucoside (2.946 mg/g) and apigenin-rutinoside (2.571 mg/g). Liquiritigenin was also present in a significant amount (1.268 mg/g) while the other compounds were found in lower, though still significant, amounts.

**Figure 1:**
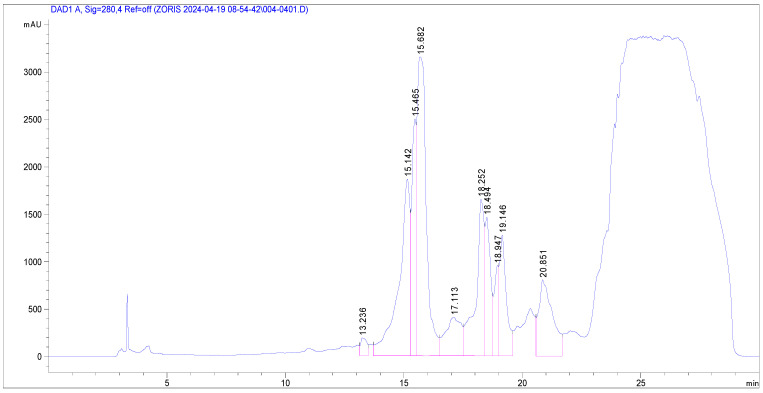
HPLC-PDA chromatograms of *G. glabra* recorded at 280 nm.

**Figure 2:**
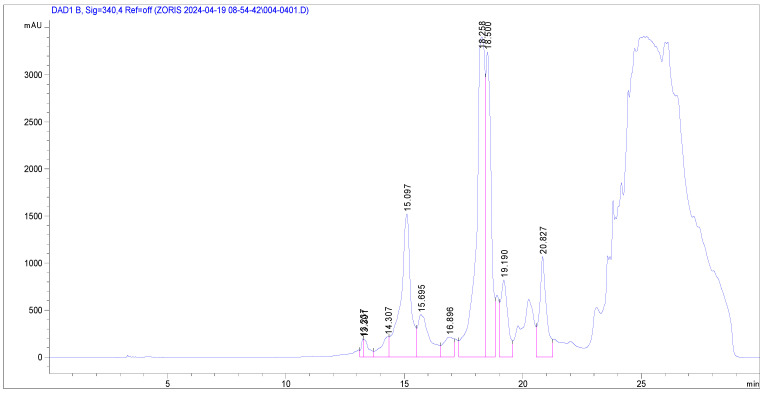
HPLC-PDA chromatograms of *G. glabra* recorded at 340 nm.

The phenolic profile of *Glycyrrhiza glabra*, obtained after methanol/water extraction and recorded at 280 and 340 nm, is shown in Figure 1 and Figure 2, respectively.

### 2.2. Total Polyphenolic Content and Radical Scavenging Activity of the Licorice Root Extract

The total phenolic content was determined using the Folin–Ciocalteu method, with gallic acid as the standard. Employing various gallic acid concentrations, the regression equation was y = 0.0104x + 0.2069, R^2^ = 0.9098. The TPC value of the licorice root extract from Romania was 169.83  ±  0.74 mg GAE/g.

**Figure 3:**
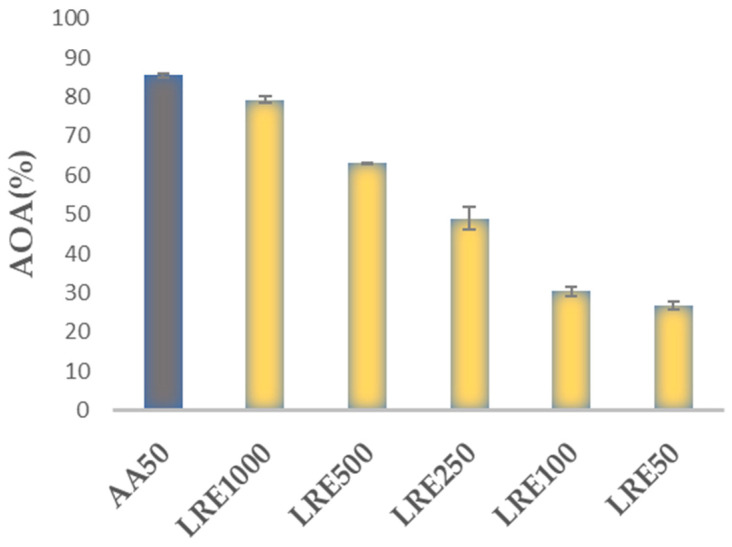
DPPH scavenging activity of LRE at various concentrations (µg/mL) and standard antioxidant ascorbic acid (50 µg/mL). Data are expressed as mean ± SD.

The antioxidant activity of the tested extract was evaluated using the free DPPH radical scavenging assay. The results showed that the tested LR methanolic extract exhibited an antioxidant ability in a concentration-dependent manner. As shown in Figure 3, higher concentrations of 1000 µg/mL had an antioxidant activity (79.29 ± 0.82%) close to that of ascorbic acid at 50 µg/mL (85.47 ± 0.62%), while lower concentrations elicited lower scavenging effects. The IC_50_, representing 50% of DPPH radical neutralization, was calculated at 385.85 ± 1.15 µg/mL (Table 2).

The mean values of the total phenolic content and IC_50_ of the DPPH free-radical scavenging activity of the licorice root extract are shown in Table 2 and Figure 3.

### 2.3. Antibacterial Activity

The antimicrobial activity of the screened methanolic extract of licorice was assessed against the following selected strains: *Streptococcus pneumoniae*, *Streptococcus pyogenes*, and *Staphylococcus aureus*, representative of Gram-positive bacteria, and *Escherichia coli* and *Pseudomonas aeruginosa*, representative of Gram-negative bacteria (Table 3). The results showed that amid the Gram-positive bacteria, the tested *Streptococcus* strains were susceptible to the tested extract, with a stronger effect on *Streptococcus pneumoniae* indicated by the LR extract MIC of 2.5 mg/mL, while the *Staphylococcus* strain presented a lower sensitivity and a MIC of 5 mg/mL. On the other hand, the Gram-negative bacteria were less sensitive to the tested extract, displaying inhibition zones of 9 mm for *Escherichia coli* and 7 mm for *Pseudomonas aeruginosa*.

### 2.4. Metal Content of the G. glabra Extract

Eleven metals were determined in the methanolic extract of *G. glabra* through atomic absorption spectroscopy. Five of them—lead, cadmium, arsenic, nickel, and cobalt—were below the detection limit (Table 4).

The concentrations of copper (4.482 ± 0.046), manganese (1.029 ± 0.030 μg/g), zinc (16.935 ± 0.364 μg/g), and chromium (19.739 ± 0.458 μg/g) were significantly below the permissible limit (20 μg/g for Cu, 900 μg/g for Mn, 100 μg/g for Zn, and 30 μg/g for Cr) according to Romanian law 756/1997.

The measured concentration of iron was 129.529 ± 7.050 μg/g. The concentration of aluminum was 557.017 ± 27.781 μg/g.

### 2.5. Irritative Potential Evaluation Using the HET-CAM Assay

The HET-CAM assay was carried out for the evaluated licorice root extract in order to estimate, in a semi-quantitative manner, the potential irritation upon topical application on epithelial tissues. The assessment was performed in vivo on the chicken embryonic egg’s chorioallantoic membrane by observing vascular events, including hyperemia, hemorrhage, and coagulation, during the 300 s after the application of the extract at a concentration of 1000 µg/mL.

**Figure 4:**
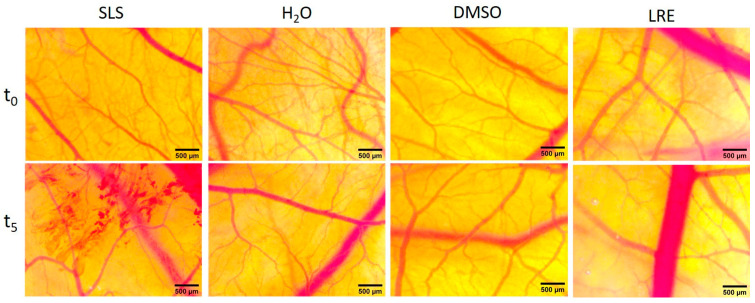
Evaluation of LRE using HET-CAM assay. Stereomicroscopic captures show aspect of chorioallantoic membrane before application (t_0_) and 5 min (t_5_) after application of licorice extract, next to the positive control (SLS), negative control (distilled H_2_O), and solvent control (DMSO); scale bars represent 500 µm.

The HET-CAM assessment showed the absence of any impairment towards vascular functionality of the chorioallantoic membrane in terms of hemorrhage, vascular lysis, and coagulability. The test involved a comparison with SLS, which induced a strong irritative effect. The tested licorice root extract, similar to the distilled water-negative control, did not induce any alteration of vascular functionality. Also, DMSO, as a solvent control, showed no irritative effect. Using the Luepke scale [34] (Luepke scale: 0–0.9—non-irritant; 1–4.9—weak irritant; 5–8.9—moderate irritant; 9–21—strong irritant), LRE corresponds to a non-irritant extract (Figure 4 and Table 5), with good tolerability and biocompatibility, thus recommendable for skin applications.

## 3. Discussion

Out of the hundreds of compounds known to be present in licorice root, there are a few that have gained more interest over the years, thanks to their antioxidant and anti-inflammatory potential, which could further be used in treating several chronic diseases and skin conditions. Most of these are phenolic compounds, while glycyrrhizin—a triterpenoid saponin—is the principal component quantitatively [35]. Phenolic compounds (including flavonoids and their derivatives) are secondary metabolites of licorice roots [36], representing some of the most studied groups of phytochemicals that are known to possess powerful antioxidant capabilities and high anti-inflammatory properties, with important roles in different mechanisms of action [37]. On the other hand, glycyrrhizin has also been extensively studied for many pharmacological properties, including anti-inflammatory and antioxidant effects, being useful for its hepatoprotective, anti-ulcerative, anti-cancer, and anti-psoriatic activity [6].

In Europe, licorice is grown for commercial use mostly in the southern, warmer regions like Italy and Spain, thanks to the favorable pedoclimatic environment for its growth [6,38]. However, due to the climatic changes in the past years, the conditions in Romania have changed and become more favorable for growing plants that normally need higher temperatures [39]. The present study is centered on the profiling of the licorice cultivated in Romania to evaluate its phytochemical, antibacterial, and antioxidant properties, which are detectable as potential sources for future pharmacological uses.

The type of extraction solvent is considered one of the main factors affecting the extraction efficiency of the bioactive constituents. The literature reports the following different solvents used for extracting glycyrrhizin and phenolic compounds: water, ethanol, methanol, chloroform, and ethyl acetate [40,41]. More studies found that methanol extraction yielded higher concentrations of these components than other types of solvents. Charpe and Rathod [41] found that ultrasound-assisted extraction gives better results than the Soxhlet extraction. Besides the fact that the necessary equipment is rather inexpensive, and the extraction times and energy consumption are low in this case, the ultrasound-assisted methods have the advantage of promoting mass transfer through decomposing cell walls, making them more efficient [42]. Considering this information, the elected extractive method for the licorice root in this study was 80% methanol as the solvent, employing ultrasonication.

### 3.1. HPLC Profiling

Because of its high sensitivity and reproducibility, it is known that high-performance liquid chromatography (HPLC) plays an important role in the determination and chemical analysis of herbal extracts and the medicine obtained from these extracts. It has been used extensively for qualitative and quantitative studies on *Glycyrrhiza* species and is still considered an essential method of analysis [11].

In the present study, the HPLC results show that the major bioactive component from the Romanian *G. glabra* extract was glycyrrhizin, having a concentration of 13.927 mg/g. Most studies present glycyrrhizin as the main component of the licorice roots, with a variable concentration between 1.212 mg/g and 516 mg/g, depending on the composition of the soil, environmental conditions, genotype, and the time when the plant was harvested [43,44,45]. However, most studies on licorice have been conducted using specimens from Iran and China, and there are only a few studies on European licorice [46]. Generally, licorice species originating from countries in the Middle East and China yield a higher number of phytochemicals than the cultures in Europe [40,47]. However, the composition and concentration of phytochemicals identified in Romanian licorice root still make it a good candidate for developing certain medications and topical preparations.

Glycyrrhizin has been extensively studied, not only for being the major constituent of licorice root but also for the number of benefits it has, either when consumed orally or applied topically [48]. Together with its metabolites, it can lower the hepatic metabolism of aldosterone, has hepatoprotective properties, can inhibit the development of certain viruses, and has been shown to have significant antioxidant and anti-inflammatory effects [49]. As far as being applied topically, glycyrrhizin and its metabolite 18β-glycyrrhetinic acid have been shown to have antimicrobial, anti-inflammatory, and antioxidative properties, being particularly useful in skin conditions like atopic dermatitis, hyperpigmentation, and androgenic alopecia [19].

Another category of secondary metabolites is represented by the polyphenolic compounds, which have an important therapeutic value, especially through their ability to trap free radicals, thus protecting various biological compounds/structures against oxidative stress. The main phenolic compounds discovered by Nomura and Fukai (1998) in licorice species include liquiritin, isoliquiritin, liquiritin apioside, coumarins, chromenes, isoprenoid-substituted flavonoids, and chromenes [40]. In the present study, the following phenolic compounds were identified: liquiritin, luteolin-rutinoside, apigenin-apiosyl-glucoside, apigenin-rutinoside, luteolin-glucoside, liquiritigenin-apiosyl-glucoside, apigenin-methylether-glucoside, and liquiritigenin. Most of these compounds have been previously studied for their effects and have been proven to manifest those effects mainly through their antioxidant capacities [50,51]. Liquiritin was the second-highest component of the analyzed *G. glabra* extract., It represents an intensively studied compound for its antioxidant and anti-inflammatory properties. In the present study, using an 80% methanolic solution, the amount of liquiritin was 5.037 mg/g. Yu et al. reported a liquiritin concentration of 1.09 ± 0.04, mg/g, which is significantly lower than the licorice in Romania. However, in Yu et al.’s study, the solvent used was 70% ethanol in water. They also found liquiritigenin (the aglycone of liquiritin) in a small quantity (0.08 ± 0.01, mg/g) while the present extract has a higher concentration of 1.268 mg/g. However, in another extensive study run on 20 different samples purchased in Taiwan, Wang found liquiritin to vary between 0.451 mg/g and 30.729 mg/g and liquiritigenin between 0.108 mg/g and 2.174 mg/g [45].

### 3.2. Total Phenolic Content and Radical Scavenging Activity of the Licorice Root Extract

In the present study, the total phenolic content assessed by the Folin-Ciocalteu method was 169.83 mg GAE/g. Many studies have described the correlation between the TPC and the antioxidant activity of different plants [36,51,52]. However, they have also observed that this correlation is not always linear, so it does not mean that a higher phenolic content will automatically yield better radical scavenging activity. There is also variability in the TPC due to other factors, like the period when the material was collected, the part that was used, and, more crucially, the climate and geographical characteristics where the plant grew [53,54]. The phenolic content of the licorice root obtained from Romania shows the presence of an important quantity of phenolic acids in the 80% methanolic extract, compared to different ethanolic extracts from Romania, which showed 63.39 mg/g [55]. Another study analyzed licorice root from Poland using methanol and ultrasonication as extraction, and the TPC was 17.55 mg/g [56], while another licorice methanolic extract from India yielded a TPC of 79.29 mg/g [57]. The majority of these studies were conducted on a type of ethanolic extract and demonstrated great variability in phenolic content, ranging from 7.88 mg/g in an extract from Egypt [58] to 185.7 mg/g in a licorice root extract from Turkey [59,60]. Our study indicates an antioxidant activity of 79.29% in the methanolic extract of the Romanian licorice root, for the higher concentration of 1000 µg/mL, which was close to the tested standard antioxidant activity of ascorbic acid (85.47%), in a concentration of 50 µg/mL.

The calculated IC_50_ for the DPPH scavenging activity of the analyzed Romanian licorice extract indicated a high value of 385.85 µg/mL, suggesting a modest antioxidant capacity of the extract, still a better effect compared to an ethanolic extract of licorice root obtained from the U.K. (805 µg/mL) [61]. This higher IC_50_ value may be explained by the lower amount of flavonols and flavanols, known to have stronger antioxidant potential [62], while our tested extract showed a higher number and significant concentrations of flavones and flavanone derivatives.

### 3.3. Antimicrobial Activity

The antimicrobial properties of various extracts of licorice, obtained from different geographical areas, have been reported throughout the years, with studies proving the importance of these properties in the future of medicine [63,64,65]. There is a wide range of results, mostly varying depending on the area from where the licorice was harvested and the extraction method, though not only [25,26,27,63]. For example, Qazi et al. show that ethanolic extracts of licorice root have greater antibacterial properties than aqueous extracts [66]. There are a few studies that report the antimicrobial activity of methanolic licorice extract, with mostly moderate results [11]. Taking into consideration a significant number of studies, it has been discussed that there is a connection between the chemical composition of the plant and the antimicrobial activity it possesses. Tanaka et al. [64] found that glycyrrhizin (the main component in licorice roots) and glycyrrhetinic acid did not exhibit antibacterial activity against pathogens like *S. pyogenes*, *H. influenzae*, and *M. catarhalis*. On the other hand, in their study, Astaf’eva et al. [26] concluded that the antibacterial activity of licorice extract is directly dependent on the amount of glycyrrhizin and 18β-glycyrrhetinic acid present in the extract. Furthermore, Thakur et al. conducted a study to specifically analyze the properties of glycyrrhizin and found that three different extracts (aqueous, ethanolic, and methanolic) possess good antibacterial activity on strains like *S. aureus* and *E. coli*, all three extracts having significant amounts of saponins and flavonoids in their content. This finding indicates that the presence of these two major classes is responsible for many of the current uses (including the antioxidant, antibacterial, and anti-inflammatory effects).

In the present study, as shown in the results section, the methanolic extract of the licorice root showed significant antibacterial inhibition for the Gram-positive tested bacterial strains. In this case specifically, activity on the Gram-negative strains was insignificant.

The tested LRE demonstrated great activity on *S. pyogenes*, and also on *S. pneumoniae*. Quazi [65] also studied the effect of licorice root extract on *S. pyogenes*, finding that both aqueous and ethanolic extracts have good antimicrobial activity on this strain but that the ethanolic extract is twice as effective [67]. Several other studies also show that licorice extract has good antibacterial activity on Gram-negative pathogens, especially on *E. coli* [25,26]. It seems that the effect on Gram-negative bacteria is greatly impacted by the dosage used [25].

### 3.4. Heavy Metals

A large part of the population considers the use of medicinal plants, as an alternative to conventional medicine, to be safer and less toxic to the human body [68]. Nevertheless, according to the World Health Organization (WHO), herbal medicines can be contaminated with natural and chemical contaminants, including heavy metals, which may be harmful to consumers. Since toxic substances are likely to be present in many foods, the concomitant ingestion of plant products would add to the total concentration of metals consumed by humans, even if best practice guidelines are followed [69]. Although the metals are of natural origin or come from anthropogenic activities, they are an integral part of the food chain. For this reason, it is essential to determine the concentration of these elements at the trace level, considering that some of them may present a high risk of toxicity for humans [70].

Trace quantities of some metal ions are essential for living organisms. Metals like copper (Cu), iron (Fe), manganese (Mn), cobalt (Co), nickel (Ni), and zinc (Zn) have a beneficial role in plant growth and development [71]. Other metals, so-called “non-essential” metals, such as lead (Pb), cadmium (Cd), and arsenic (As) have no specific biological functions in an organism, but they are toxic at low concentrations, persistent, and accumulate through food consumption [72]. The limits proposed by the National Sanitation Foundation for these toxic metals in finished dietary supplements are 0.02 mg/day for Pb, 0.006 mg/day for Cd, and 0.01 mg/day for As [69].

In this study, eleven metals, some known for their toxic potential, were scanned and quantified in licorice root dry extract. Five potentially toxic heavy metals (lead, cadmium, arsenic, nickel, and cobalt) were below the limit of detection. Therefore, from this point of view, the use of Romanian licorice root extract as a therapeutic remedy can be considered safe.

For the other six analyzed elements, the following concentrations were found: 1.029 μg/g (the equivalent of ppm and mg/kg) for manganese (Mn), 557.017 μg/g for aluminum (Al), 129.529 μg/g for iron (Fe), 4.48 μg/g for copper (Cu), 16.9 μg/g for zinc (Zn), and 19.7 μg/g chromium (Cr). All of these values are below or within the allowed limits indicated by the WHO [69].

### 3.5. HET-CAM Irritation

To further characterize the LR methanolic extract from Romania, we assessed the potential toxicity exhibited on epithelial tissues by using an in vivo model that employs the chorioallantoic membrane of the chick embryo. There is a lack of data regarding the potential irritability of licorice extracts upon topical application. A single study tested a hair tonic containing licorice extract from Indonesia, and results showed that at the used concentration of 2.5%, the formulation had a certain degree of topical toxicity; still, the study did not include samples with only licorice roots, and authors indicate a possible effect of the other ingredients [73]. Another evaluation of a hydrogel containing 5% extract was used in a clinical study that concluded that the tested formulation accelerates the wound healing properties [74].

Therefore, the in vivo evaluation of the Romanian licorice root extract is even more valuable as a characteristic of its good tolerability on epithelial living tissues. The tested extract lacked irritability potential even at a concentration of 1000 µg/mL.

Our study evaluated an 80% methanolic extract of licorice root obtained from Romania as an easily available, locally accessible resource. As compared to numerous other studies, the present evaluation showed that the methanolic extract has significant amounts of glycyrrhizin (13.927 mg/g), the triterpenic saponin considered to be the major phytochemical in *G. glabra*, alongside significant amounts of flavonoid-type phenolic compounds, including major constituents such as liquiritin, liquiritigenin-apiosyl-glucoside, and apigenin-rutinoside; flavonoids with highly valuable biological properties, beneficial in different pathologies.

Moreover, our work produces significant data regarding the metal content of the evaluated licorice root extract, with an important focus on safety issues. Results showed that the heavy metal count was below the detectable limit, while other microminerals were present in small concentrations, less than the regulated upper limits. Finally, the HET-CAM assessment showed no irritability of the extract, rendering it safe for topical applications. All these data validate the supposition that *G. glabra* extract cultivated in Romania represents a good, safe alternative local source of both glycyrrhizin and phenolic compounds, with the absence of dangerous heavy metals. Additionally, the reported biological data, presented by the antioxidant capacity, along with the good in vivo tolerability evaluated on epithelial tissues, complete the characteristics of the Romanian licorice root extract as a source of the triterpenic glucoside glycyrrhizin, as well as of polyphenolic compounds, especially flavonoids such as liquiritin and liquiritigenin-apiosyl-glucoside, recommending the extract for its potential therapeutic effects, with multiple medical applications.

## 4. Materials and Methods

### 4.1. Plant Material and Preparation of Licorice Root Extract

The licorice root (*Liquiritiae radix*) was purchased from Fares, a Romanian producer of plant-based remedies, in October 2023. The dried plant material was authenticated by the Department of Pharmacognosy, and a voucher specimen (23-GG-CD-2023) was deposited in the Herbarium of the Department of Pharmacognosy, Faculty of Pharmacy, “Victor Babes” University of Medicine and Pharmacy, Timisoara.

The licorice roots were ground with a mechanical grinder. The extraction technique was selected and adapted to our laboratory facilities to optimize extraction for triterpenic and polyphenolic compounds from licorice [75]. A methanolic extract was prepared using the following: 10 g powder and 100 mL MeOH/H_2_O (*v*/*v*-80/20) for ultrasonication (60 min, 50 °C, 59 KHz) [66]. The product obtained was then filtered and evaporated by Rotavapor 50 °C, 150 rpm, pressure 250, followed by drying at 50 °C.

### 4.2. HPLC-DAD-ESI+ Determination of Phytocompounds

#### 4.2.1. Sample Preparation

The dried extract was solubilized in 1 mL ethanol of 96% purity. The probe was vortexed by a Heidolph Reax top for 1 min, then transferred into the ultrasound bath Elmasonic E 15 H for 30 min. The extract was filtered through a 0.45 µm Chromafil Xtra nylon filter, and 20 µL was injected into the HPLC system.

#### 4.2.2. Chromatographic Conditions

The Agilent 1200 HPLC system, equipped with a quaternary pump, solvent degasser, autosampler, and a UV–VIS detector with a photodiode (DAD) coupled with a single quadrupole mass detector (MS) Agilent model 6110 (Agilent Technologies, Santa Clara, CA, USA) were used.

The separation of the compounds was carried out on a Kinetex XB C18 column, dimensions 4.6 × 150 mm^2^, with 5 μm particles (Phenomenex, Torrance, CA, USA), using the mobile phases (A) water + 0.1% acetic acid and (B) acetonitrile + 0.1% acetic acid in the gradient below, for 30 min at a temperature of 25 °C, with a flow rate of 0.5 mL/min. Gradient (expressed in % B): 0 min, 5% B; 0–2 min, 5% B; 2–18 min, 5%–40% B; 18–20 min, 40%–90% B; 20–24 min, 90% B; 24–25 min, 90%–5% B; 25–30 min, 5% B. The spectral values were recorded in the 200–600 nm range for all peaks.

For the MS, a full scan ESI positive ionization mode was used in the following working conditions: capillary voltage 3000 V, temperature 350 °C, nitrogen flow 7 L/min, and *m*/*z* 120–1200. Data acquisition and the interpretation of results were carried out using Agilent ChemStation software, version B.02.01 SR2.

The phenolic compounds were identified by comparing the retention time, UV–VIS spectral absorption, and mass with those of the standard compounds and with the data from the specialized literature.

The quantification of phenolic compounds was conducted for flavanones and flavones using the calibration curve made with luteolin. Measurements were performed in triplicate. The results were provided as mean ± SD (standard deviation).

#### 4.2.3. Chemical Reagents and Materials

Acetonitrile, of HPLC purity, was purchased from Merck (Darmstadt, Germany) and ultrapure water was purified with the Direct-Q UV system from Millipore (Burlington, MA, USA). Standard luteolin (99% HPLC purity) was supplied from Sigma (Livonia, MI, USA).

### 4.3. Total Phenolic Content Determination

To estimate the total phenolic content of the *Glycyrrhiza glabra* root extract (GGRE), the Folin–Ciocâlteu technique was used [76]. Diluted solutions of the dried extracts (1000 µg/mL) were added to tubes containing Folin–Ciocâlteu’s phenol reagent previously diluted (1:10), and sodium carbonate solution (Na_2_CO_3_ 75 g/L) was added after 5 min at room temperature. The vortexed mixed solution was allowed to rest at room temperature for 2 h in the dark. Subsequently, the absorbance was measured at 760 nm using a UV–VIS spectrophotometer (T80+, PG Instruments Ltd., London, UK). Gallic acid (0–200 µg/mL) was employed to obtain the calibration curve, and the estimation of the phenolic content was expressed as mg of gallic acid equivalents (GAE)/g of dry extract (DE). The measurements were carried out in triplicate.

### 4.4. Free Radical Scavenging Capacity Using the DPPH Assay

The antioxidant activity of *Glycyrrhiza glabra* root extract was estimated using the stable radical 2,2-diphenyl-1-picrylhydrazyl (DPPH) based on a change in color from purple to yellow and a decrease in absorbance when mixed with a hydrogen-donating antioxidant [77,78,79]. Briefly, various concentrations (50–1000 µg/mL) of the GGRE extract, in volumes of 0.2 mL, were added to 1.8 mL of freshly prepared 0.1 mM DPPH in methanol. Absorbance was measured against blank samples at 517 nm using an UV–VIS spectrophotometer (T80+, PG Instruments Ltd., London, UK), after an incubation of 30 min in the dark at room temperature. Ascorbic acid (AA) was used as the control standard antioxidant. The free radical scavenging capacity of the extract was indicated by a decrease in the registered absorbance, and the DPPH radical’s percentage of inhibition was calculated as antioxidant activity in percentages using the following equation:

$$AOA\left(\%\right)=\left[\frac{A_{0}-A_{s}}{A_{0}}\right]\times 100$$

where *A*_0_ = absorbance of the blank sample and *A*_s_ = absorbance of the tested samples.

### 4.5. Antimicrobial Activity Assay

Five bacterial strains from the American Type Culture Collection were employed for the antimicrobial activity testing. The selected species are illustrative of human pathogenic bacteria. The strains included in the study were *Staphylococcus aureus* ATCC 29213, *Streptococcus pyogenes* ATCC 19615, *Streptococcus pneumoniae* ATCC 49619, *Escherichia coli* ATCC 25922, and *Pseudomonas aeruginosa* ATCC 27853 (Microbiologics, Saint Cloud, MN, USA). All the tested bacteria were isolated on Columbia agar with 5% sheep blood (Oxoid, Hampshire, UK).

The initial stage of the antimicrobial activity testing involved a disk diffusion assay following the European Committee on Antimicrobial Susceptibility Testing (EUCAST) and the Clinical Laboratory and Standard Institute (CLSI) guidelines and was extensively described in our previous studies [80,81,82]. The positive controls were levofloxacin (5 µg) disks (BioMaxima, Lublin, Poland), while a blank paper disk served as the negative control. The results of the sensitivity testing for levofloxacin fell within the ranges recommended by the EUCAST and CLSI standards.

To determine the minimum inhibitory concentration (MIC), the standardized bacterial inoculum of 0.5 McFarland was diluted in NaCl 0.85% (bioMérieux, Marcy-l’Étoile, France) to obtain a concentration of approximately 5 × 10^5^ colony-forming units/mL (CFU). Subsequently, the bacterial suspension and the tested extract were added to Mueller–Hinton broth (Oxoid, Hampshire, UK), supplemented with blood and β-NAD for Streptococcus species, resulting in a series of dilutions with concentrations of 5, 2.5, and 1.25 mg/mL. Following a 24 h incubation period at 35 °C, the lowest concentration exhibiting no visible growth was deemed to be the MIC value. To serve as a control for bacterial viability, 0.5 mL of Mueller–Hinton broth was added to a tube containing 0.5 mL of microbial suspension. Each determination was performed in triplicate for each tested strain.

### 4.6. Metal Analysis by Atomic Absorption Spectroscopy (AAS)

About 0.1 g of the dried extract was treated with 5.0 mL 67% HNO_3_ (Sigma Aldrich, Jena, Germany) in high-pressure Teflon vessels DAP-60K and subjected to microwave acidic digestion in a three-step program (Table 6) using the microwave system MWS-2 (Berghof, Eningen, Germania). After digestion, the solid part was removed by filtration, and the samples were brought to a volume of 20 mL with ultrapure water (EASYpure RoDi^®^ apparatus, Barnstead, NH, USA).

The concentrations of detected metals were determined using a spectrophotometer novAA 400G (Analytik Jena, Jena, Germany) equipped with a graphite furnace, an auto sampler MPE60, and a Cookbook for all elements. Sample analysis and data processing were performed with the WinAAS 3.17.0 software. If the metal concentration was too high (above the upper limit of the calibration curve), the samples were properly diluted with 0.5% HNO_3_ before injection. Each determination was performed in six replicates.

For each element, a calibration curve at the specific wavelength was previously registered (Table 7). The calibration solutions were prepared with CertiPUR^®^ standard solution (1000 mg/L, Merck, Darmstadt, Germany) by dilution with ultrapure water (Barnstead, EASYpureRoDi^®^ apparatus).

### 4.7. Irritative Potential Using the HET-CAM Assay

In order to establish the potential irritability of *Glycyrrhiza glabra* root extract, the HET-CAM assay was employed. The method is known as an alternative protocol for the evaluation of the potential irritative effect of samples proposed for ophthalmic and dermatological use, as well as to establish in vivo biocompatibility [83,84,85]. The protocol involves the observation of possible undesirable changes happening to the chorioallantoic membrane of chick embryos upon exposure to the test solution. The standard procedure involves fertilized chicken (*Gallus gallus domesticus*) eggs incubated in a controlled humidified atmosphere at 37 °C, prepared by performing a resealable opening on the upper shells to reveal the highly vascularized chorioallantoic membranes [33,85].

On day 10 of incubation, 300 µL of the *Glycyrrhiza glabra* root extract was applied on the vascularized CAM, monitoring, by means of a stereomicroscope for 300 s, the potential atypical changes, including hemorrhage, lysis, and coagulation. The irritability score (IS) was calculated using the first appearance of the mentioned events, using the following equation:

$$IS=5\times \left[\frac{301-Sec\;H}{300}\right]+7\times \left[\frac{301-Sec\;L}{300}\right]+9\times \left[\frac{301-Sec\;C}{300}\right],$$

where Sec H (hemorrhage) = first appearance (in seconds) of bleeding vessels, Sec L (lysis) = first appearance (in seconds) of vessel disintegration of the vessel, and Sec C (coagulation) = first appearance (in seconds) of vessel-related protein denaturation.

The study also included testing a positive control, a 1% *w*/*v* solution of sodium lauryl sulfate (SLS); a negative control involved treatment with distilled water (H_2_O) and a solvent control, dimethyl sulfoxide (DMSO), in a concentration of 0.5% *v*/*v*. All results were classified according to Luepke’s scale as 0–0.9—non-irritant, 1–4.9—weak irritant, 5–8.9—moderate irritant, and 9–21—strong irritant [34], which allowed us to identify the type of irritant for each sample. The investigation was carried out using stereomicroscopy (ZEISS SteREO Discovery.V8, Göttingen, Germany), while image acquisition and processing were performed using Axiocam 105 color and AxioVision SE64. Rel. 4.9.1 Software, (ZEISS, Göttingen, Germany), ImageJ (ImageJ Version 1.54i, https://imagej.nih.gov/ij/index.html (accessed on 23 September 2024)), and GIMP software (GIMP 2.10.36 revision 1, https://www.gimp.org/ (accessed on 23 September 2024)) [33]. The experiment was performed in triplicate.

## 5. Conclusions

*G. glabra* L. has been the focus of many researchers throughout the years because of its great potential to treat many chronic diseases. Standardization and safety are essential in asserting the desired therapeutic effect when it comes to any type of herbal extract. This report provides relevant information about the composition and quality control of an 80% methanolic extract of licorice root obtained from Romania. The HPLC showed that the methanolic extract is rich in glycyrrhizin (13.927 mg/g), the triterpenic saponin considered to be the major phytochemical in *G. glabra*, while liquiritin, liquiritigenin-apiosyl-glucoside, and apigenin-rutinoside were best represented among the other identified phenolic compounds. The ability to scavenge free radicals was also measured, with a high antioxidant activity, close to ascorbic acid at 1000 µg/mL. The antibacterial assay revealed significant activity on Gram-positive strains, such as *S. pyogenes* and *S. pneumoniae*. The safety profile of the extract was evaluated, indicating that all tested heavy metals were below the detectable limit, while other elements were present in small concentrations. Lastly, the HET-CAM investigation showed that the methanolic licorice extract possesses no irritability on the chorioallantoic membrane.

All these data validate the supposition that the *G. glabra* extract obtained from Romania represents a good source of both glycyrrhizin and phenolic compounds, with significant antioxidant and antibacterial activity. It is also safe from a toxicologic standpoint and demonstrates good in vivo tolerability. For these reasons, it can be further considered for biological studies and therapeutic applications.

## Figures and Tables

**Table 1:** Identification and quantification of phenolic and triterpenic compounds in the licorice root extract (LRE) expressed as mg/g dry extract (d.e.). R_t_—retention time; [M + H]^+^—molecular ion.

Peak No.	R_t_ (min)	UV λ_max_ (nm)	[M + H]^+^ (*m*/*z*)	Compound	Class	LRE (mg/g d.e.)
1	13.27	340, 260	595, 287	Luteolin-rutinoside	Flavone	0.231 ± 0.038
2	14.19	341, 256	565, 271	Apigenin-apiosyl-glucoside	Flavone	0.295 ± 0.022
3	15.06	341, 256	579, 271	Apigenin-rutinoside	Flavone	2.571 ± 0.075
4	16.84	340, 260	449, 287	Luteolin-glucoside	Flavone	0.471 ± 0.007
5	19.01	340, 260	463, 287	Luteolin-methylether-glucoside	Flavone	0.393 ± 0.005
6	19.19	341, 256	447, 271	Apigenin- methylether-glucoside	Flavone	0.996 ± 0.026
7	18.25	360	418, 257	Liquiritin	Flavanone	5.037 ± 0.054
8	18.49	360	551, 418, 257	Liquiritigenin-apiosyl-glucoside	Flavanone	2.946 ± 0.046
9	20.85	360	257	Liquiritigenin	Flavanone	1.268 ± 0.064
10	15.67	254	823	Glycyrrhizin	Triterpenic saponin	13.927 ± 0.016

**Table 2:** Total phenolic content (TPC) and IC_50_ for DPPH scavenging activity of LRE.

Extract	TPC (mg GAE/g d.e.)	DPPH Scavenging Activity (IC_50_ µg/mL)
LRE	169.83 ± 0.74	385.85 ± 1.15

**Table 3:** Antibacterial activity of LRE, as inhibition diameters (mm) and minimal inhibitory concentration (MIC), for selected strains after incubation with extract.

Microbial Strains	Disk Diffusion Method (Inhibition Zones in mm)	MIC (mg/mL)
*Streptococcus pneumoniae* ATCC 49619	17	2.5
*Streptococcus pyogenes* ATCC 19615	16	5
*Staphylococcus aureus* ATCC 25923	13	5
*Escherichia coli* ATCC 25922	9	NA
*Pseudomonas aeruginosa* ATCC 27853	7	NA

**Table 4:** The concentration of metals in licorice root extract.

Sample	Element Concentration (μg/g) *										
	Fe	Cu	Ni	Mn	As	Al	Zn	Co	Pb	Cr	Cd
LR extract	129.529 ± 7.050	4.482 ± 0.046	ND **	1.029 ± 0.030	ND	557.017 ± 27.781	16.935 ± 0.364	ND	ND	19.739 ± 0.458	ND

* Mean of six determinations ± standard deviation. ** ND: not detected (below limit of detection).

**Table 5:** Irritation scores and type of effect induced by LRE in HET-CAM assay.

Samples	Irritation Score	Type of Effect
SLS	17.19 ± 0.24	strong irritant
H_2_O	0 ± 0	non-irritant
DMSO	0 ± 0	non-irritant
LRE	0 ± 0	non-irritant

**Table 6:** Program parameters (T—temperature, t—time, p—power) for microwave acidic digestion.

**Digestion Parameters**	**T1**	**t1**	**p1**	**T2**	**t2**	**p2**	**T3**	**t3**	**p3**
	160 °C	15 min	80%	210 °C	15 min	90%	Gradual decrease in temperature	15 min	0

**Table 7:** Operating conditions for the metal analysis by atomic absorption spectroscopy.

No	Metal	Wavelength, λ (nm)	Calibration Range, (μg/L)	Calibration Curve Abs = f(conc.)	R^2^
1	Fe	248.3	0–14.4	y = 0.021707 + 0.010187x	0.9988
2	Cu	324.8	0–18.0	y = 0.038506 + 0.048577x	0.9931
3	Ni	232.0	0–31.5	y = 0.115634+ 0.006821x	0.9998
4	Mn	279.5	0–3.36	y = 0.007792 + 0.112496x	0.9925
5	As	193.7	0–52.8	y = −0.001185 + 0.001544x	0.9927
6	Al	309.3	0–52.8	y = 0.006978 + 0.001749x	0.9971
7	Zn	213.9	0–8.0	y = 0.071658 + 0.092202x	0.9827
8	Co	240.7	0–21.6	y = 0.007448 + 0.008841x	0.9974
9	Pb	283.3	0–38.0	y = 0.004606 + 0.004331x	0.9959
10	Cr	357.9	0–20.0	y = 0.013314 + 0.018746x	0.9932
11	Cd	228.8	0–2.0	y = 0.007384 + 0.100405x	0.9903

## Data Availability

The data supporting the findings of the study are available within the article.
